# Automated Detection of Hypertension Using Continuous Wavelet Transform and a Deep Neural Network with Ballistocardiography Signals

**DOI:** 10.3390/ijerph19074014

**Published:** 2022-03-28

**Authors:** Jaypal Singh Rajput, Manish Sharma, T. Sudheer Kumar, U. Rajendra Acharya

**Affiliations:** 1 Department of Electrical and Computer Science Engineering, Institute of Infrastructure Technology Research and Management, Ahmedabad 380026, India; jaypal2020@gmail.com (J.S.R.); Sudheer.kumar.20pE@iitram.ac.in (T.S.K.); 2 Department of Electronics and Computer Engineering, Ngee Ann Polytechnic, Singapore 639798, Singapore; aru@np.edu.sg; 3 Department of Bioinformatics and Medical Engineering, Asia University, Taichung 41354, Taiwan; 4 Department of Biomedical Engineering, School of Science and Technology, Singapore 639798, Singapore

**Keywords:** hypertension, BCG signal, hypertension BCG signal classification, deep learning, convolutional neural network

## Abstract

Managing hypertension (HPT) remains a significant challenge for humanity. Despite advancements in blood pressure (BP)-measuring systems and the accessibility of effective and safe anti-hypertensive medicines, HPT is a major public health concern. Headaches, dizziness and fainting are common symptoms of HPT. In HPT patients, normalcy may be observed at one instant and abnormality may prevail during a long duration of 24 h ambulatory BP. This may cause difficulty in identifying patients with HPT, and hence there is a possibility that individuals may be untreated or administered insufficiently. Most importantly, uncontrolled HPT can lead to severe complications (stroke, heart attack, kidney disease, and heart failure), mainly ignoring the signs in nascent stages. HPT in the beginning stages may not present distinct symptoms and may be difficult to diagnose from standard physiological signals. Hence, ballistocardiography (BCG) signal was used in this study to detect HPT automatically. The processed signals from BCG were converted into scalogram images using a continuous wavelet transform (CWT) and were then fed into a 2-D convolutional neural network model (2D-CNN). The model was trained to learn and recognize BCG patterns of healthy controls (HC) and HPT classes. Our proposed model obtained a high classification accuracy of 86.14% with a ten-fold cross-validation (CV) strategy. Hence, this is the first use of a 2D-CNN model (deep-learning algorithm) to detect HPT employing BCG signals.

## 1. Introduction

When there is increased arterial blood pressure in an individual, the condition is medically referred as hypertension (HPT) [1]. In such individuals, the pumping of oxygenated blood through the body proves a laborious task for the heart. HPT is clinically categorized into one of three categories: mild, moderate or average, and severe [1,2]. Usually, normal systolic blood pressure is 120–139 mmHg, and diastolic blood pressure is 80–89 mmHg. Mild stage HPT individuals present a systolic blood pressure of 140–159 mmHg and diastolic blood pressure of 90–99 mmHg [2,3].

Moderate or average stage HPT individuals exhibit a systolic pressure of 160–179 mmHg and a diastolic pressure of 100–109 mmHg, while individuals with more than 180 mmHg systolic and 110 mmHg diastolic blood pressures are classified as being in a severe stage of HPT. Common HPT pathological symptoms include changes in vision, headaches, dizzy spells, etc. Prolonged HPT leads to an increase in cardiovascular, neural, and renal diseases [1,4].

As per a World Health Organization (WHO) study, the number of individuals suffering from HPT globally exceeds 1.3 billion, and every 1 in 5 people have some form of controlled HPT [1,5]. The statistics of WHO report also states that HPT ranks third in the most common causes of death, and nine million people die every year due to this condition [3]. Uncontrolled HPT may induce heart muscle hypertrophy and, consequently, heart failure [3]. For the diagnosis of HPT, the standard procedure is to record 24 h of ambulatory blood pressure [1]. This process consumes time and requires sufficient expertise. To negate these, HPT is often detected using computational intelligence-based diagnostic techniques [3,6,7].

## 2. Cardio-Mechanical Signals

### 2.1. Seismocardiography (SCG) Signal

SCG measures the mechanical vibrations of the heart by embedding a sensor or leads on the chest surface of the human body [8,9]. By attaching a low-noise accelerometer to the chest, the SCG can be easily recognized. Mechanical activities in the heart, such as cardiac muscle contractions, valve movement, blood flow turbulence, and momentum changes, are thought to be the source of SCG signals [8,9]. Therefore, the properties of SCG signals are anticipated to provide helpful details that correlate with pathological or physiological events in the cardiovascular system.

### 2.2. Forcecardiography (FCG)

The chest wall vibrations caused by the mechanical activity of the beating heart are measured using the novel forcecardiography (FCG) method [10,11]. This method can acquire a low-frequency signal component that conveys essential information about the heart’s empty dynamics and ventricular filling. FCG is obtained by attaching force sensors to a patient’s chest wall [10,11]. The force-sensitive resistors are used in FCG sensors, which have previously been used to monitor muscle contractions and recognize various hand motions. This appears suitable for measuring the tissue motion generated due to multiple types of physiological mechanical events; potentially including respiration monitoring. For example, when appropriately connected to a subject’s chest, an FCG sensor can monitor the force exerted by ribcage expansions and the subsequent releases during breathing activities, allowing a respiration-related signal to be recorded alongside the FCG [10,11].

### 2.3. Gyrocardiography (GCP)

Gyrocardiography is a non-invasive technology that uses a tri-axial gyroscope sensor. In response to the heart’s activity, the GCP detects three-dimensional angular velocities of the chest [12,13,14]. Gyrocardiogram is the name given to the signal obtained during gyrocardiography. A gyrocardiogram is a low-frequency mechanical signal with a frequency range of 1–20 Hz and is measured in degrees per second [12,13,14].

The application of GCP is as follows: heartbeat detection, HRV analysis, atrial fibrillation, myocardial infarction, heart failure, and sleep monitoring. However, GCP has certain limitations. There is no generally acknowledged waveform description benchmark. There are discrepancies in the understanding of the GCG signals’ link to heart motion. The recording of the signals can be affected by motion noise. The GCP recordings may have interpersonal variation due to body mass index, age, sex, and health status. As a result, this can produce diverse beat morphologies.

As a concluding remark, the GCP, SCG, and FCG signals are widely used for recording the mechanical activity of the heart based on the sensors. However, to the best of our knowledge, HPT detection from these signals data is not available in the existing literature. Furthermore, these signals are not directly correlated with the blood pressure measurements, which will be important in HPT diagnosis. Hence, we move forward with HPT detection using BCG signals.

### 2.4. Ballistocardiography (BCG) Signal

BCG is a measurement technique that records the whole-body recoil forces (or the related displacements/velocities/accelerations) due to blood circulation. It is a novel, non-invasive-based diagnostic method used for HPT detection [4,15,16]. Micro body vibrations occur in the body due to the heart’s physiological activity, which is then picked up by the BCG to generate precise physiological signals [15,17,18,19]. These signals are generated from the reaction of the whole body from the displacement, velocity or acceleration of blood arising from the pumping action of the heart [15].

As a result, the BCG signal arises from a multitude of forces related to the cardiac movement and the blood pumped into the arteries as well as blood within the heart itself [15]. As BCG signals contain information about cardiac activity, this method has been successfully used to detect a wide variety of cardiovascular ailments [15]. Repeated motions are caused when blood accelerates rapidly in the blood vessels during systole and diastole cycles [15,16].

BCG recognizes and analyses these repeating vibrations, providing usable information about the heart and blood volume moving during a pumping cycle [16]. During the atrial systolic cycle, the center of mass of the entire body shifts towards the head as a result of blood being pumped into the blood vessels [16]. Conversely, during the atrial diastolic cycle, the center of mass of the body shifts to the peripheries [16]. A BCG waveform is generated by this center of mass shift and by heart activities known as normal respiratory cycles.

**Figure 1:**
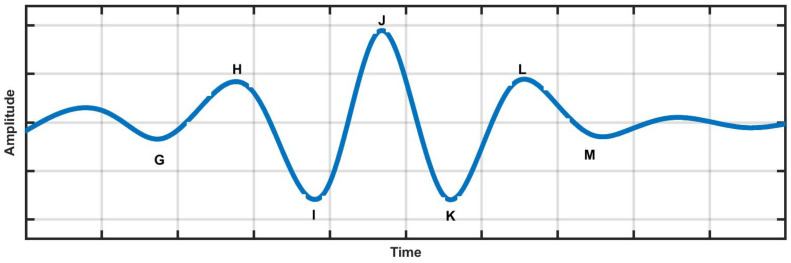
Normal BCG signal.

Figure 1 shows these waveform patterns, which are subsequently evaluated as the blood flow varies throughout a cardiac cycle [16]. Without the assistance of specialist staff, BCG sensors can be installed in residences, thereby, playing a positive role within the e-health setup [16]. A subject’s stress and emotion levels can be reduced during periods of checkup, which can lead to better attention responses. A sample BCG signal is illustrated in Figure 1, where the individual letters are assigned to the different waveforms [15,16]. The details of the BCG signal terminology are outlined in Table 1.

There is evidence in the literature that BCG analysis with machine learning could support the automated diagnosis of HPT [15,21]. BCG signals are also widely used for continuous blood pressure monitoring of the HPT subjects. BCG is a contact-based approach, as it requires the subjects to stay on suspended beds or electronic weightscales.

### 2.5. BCG Signal Related Literature

Chen et al. [22] used a linear regression technique to extract BP from the BCG signals. SBP had a statistical (mean and standards) deviation error of 9 and −5, while DBP had 1.8 and −5. In addition to this, Lee et al. [23] used ANN to diagnose the SBP and DBP from the BCG signal. The SBP and DBP had mean and standard deviation errors of 0.012±6.75 and 0.053±5.83.

Furthermore, Seok et al. [24] employed CNN to detect BP using BCG signals. During rest and exercise, the two-channel BCG signal was recorded. As a result, the authors obtained a better performance for the rest position than the exercise.

Using a Naive–Bayes classifier, Song et al. [25] classified BCG signals into HC and HPT classes with 74.5% accuracy. In addition to this, the authors extracted the heart rate variability (HRV) from BCG signals. Liu et al. [15] employed HRV signals to detect HPT and HC BCG signals and achieved an accuracy of 84.5%. Linear, non-linear, frequency, and time-domain features were extracted from HRV signals. The decision tree and support vector machine-learning classifier were used to classify HPT and HC HRV signal classes [15].

In the literature, the detection of HPT has been based on HRV signals extracted from BCG signals. Therefore, significant contributions are required in detecting the HPT from direct BCG signals with higher accuracy.

### 2.6. Diagnosis of HPT from ECG, Photoplethysmogram (PPG), and HRV Signals

A recent study by Rajput et al. [3] employed low, high-risk HPT and HC using ECG signals. The study used the wavelet decomposition method and acquired sample and wavelet entropy features. The ensemble bagged tree machine-learning classifier yielded a high classification accuracy of 99.95% with a ten-fold CV method. A convolutional neural network (CNN) architecture was developed by Soh et al. [5] for automated classification of normal and HPT ECG signals. This architecture yielded 99.9% accuracy, 100% sensitivity, and a specificity rate of 99.97%.

In three separate studies conducted by Liang et al. [26,27,28], HPT was detected using PPG signals and obtained the highest classification F-score of 94.84%. A more recent study by Jain et al. [1] used a deep-learning model, which classified ECG signals into normal, low-risk, and high-risk HPT, and a 99.68% accuracy level was achieved.

Another recent study by Alkhodari et al. [29] classified low-risk and high-risk HPT HRV signals, which were extracted from ECG signals, and a high classification accuracy of 97.08% was obtained using CNN model. Soh et al. [30] used ECG signals for categorizing HPT and normal subjects with the help of derived non-linear features and applied machine-learning techniques. In addition to this, the obtained ECG signals were subjected to five-level empirical mode decomposition (EMD) to obtain an accuracy of 97.7%.

Rajput et al. [2] also used ECG signals to develop a HPT diagnosis index (HDI), which was then used to classify HPT individuals into low risk and high risk categories. From the orthogonal wavelet filter bank, fractal dimensions and log energy features were derived and used in the study. In a study performed by Ni et al. [31], HRV signals were obtained from ECG signals and machine-learning algorithms were used to develop a multi scale fine grained method to assess the severity of HPT with an accuracy of 95%.

Melillo et al. [32] fed HRV signals to a random forest classifier, which then identified HPT patients as high risk with a predictive accuracy of 87.8%. In another recent study, Poddar et al. [33] used a support vector machine (SVM) classifier with linear and non linear features to develop an automated system using HRV signals. This system categorized HPT patients with an accuracy of 96.7%.

From the literature, it is evident that more work is required for detection of HPT using BCG signals. We present an automated HPT diagnosis using BCG signals. To the best of our knowledge, this is the first study proposed for the automated detection of HPT from BCG signals. Furthermore, to normalize the amplitude of the BCG signal, we used the z-score normalization method. After this, we segmented long length (13 h) BCG signals into 30 s epochs.

We converted each 1D-BCG (segmented 30 s epochs) signal into 2D images called scalograms using continuous wavelet transform (CWT). These scalograms are of the size 224 × 224 and are then resized to 32 × 32 and fed as input to the 2D-CNN model. Subsequently, we designed a 24-layer CNN model, which includes convolution, pooling, dense layers, dropout layers, and L1-regularization to prevent overfitting.

Hence, this study incorporates a model that allows for faster computation. CNN models, when compared to other models, are predominantly used for tasks, such as image identification and signal categorization, object and face recognition [34,35]. The proposed 2D-CNN model obtained 86.14% classification accuracy with the ten-fold cross-validation strategy.

The remaining sections of the paper are organized as follows: The Data base and methods employed are described in Section 3, and the results are given in Section 4. The results are discussed in Section 5, and the proposed work is summarized in Section 6.

## 3. Methodology

**Figure 2:**
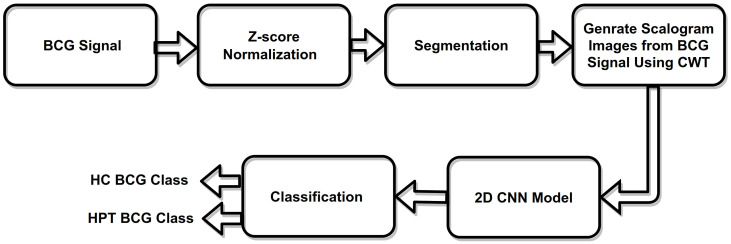
Work flow of the proposed method.

The study protocols and procedures were approved by the Northwestern Polytechnical University’s Medical Experimental Ethical Inspection Institute (No. 20170078), China. In order to normalize the amplitude of the BCG signal, we first downloaded the open-source BCG database and used the z-score normalization method. After this, we segmented the BCG signal into 30 s epochs. Then, we converted each 1D-BCG signal into 2D images called scalograms using CWT. These scalograms were then fed as the input to the 2D-CNN model. Figure 2 depicts the proposed strategy.

### 3.1. Database

**Figure 3:**
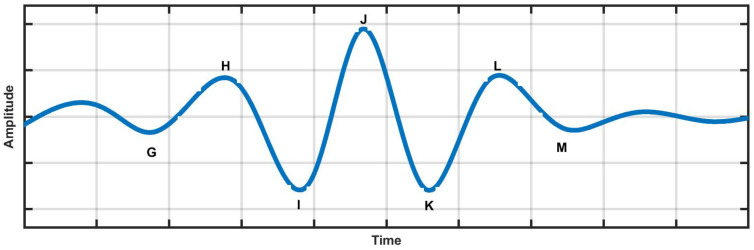
Normal (HC) BCG signal obtained from the database. The details of the G, H, I, J, K, L, and M waves of the BCG signal are mentioned in Table 1.

**Figure 4:**
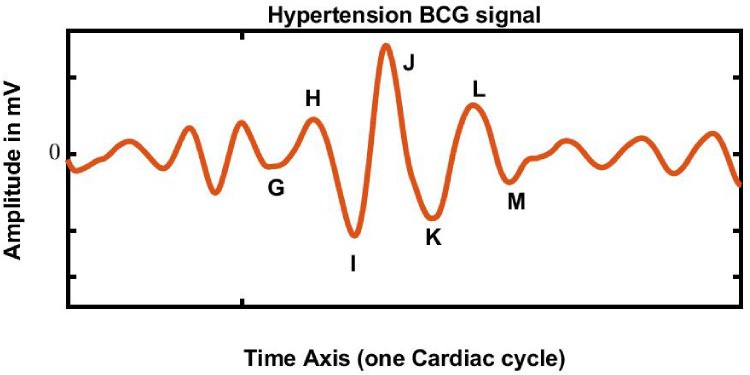
HPT BCG (abnormal) signal acquired from the database. The HPT BCG signals have fast variations in the time axis and abrupt rises and falls in amplitude compared with the normal BCG signals.

As described in Liu et al. [15], we collected 67 HC and 61 HPT BCG signals. The BCG signal sampling frequency was tuned to 100 Hz to acquire the optimum waveforms. The long length of approximately 13 h of BCG signals obtained from the study was segmented into 30 s durations. Hence, a total of 61,525 HPT segments and 71,413 HC BCG segments were obtained. A total of 132,938 BCG signal segments were used for the HC and HPT classes. The HC and HPT BCG signals obtained this study were stored in a database [15,21] and examples are shown in Figure 3 and Figure 4, respectively. The vital statistics of the participants in the study are described in [15]. Moreover, the BCG signal database was obtained from [15,21].

### 3.2. Z-Score Normalization

The amplitude scaling problem commonly observed in physiological signals (BCG database) can be overcome by performing Z-score normalization on the 13 h (long-length) database. The Z-score normalization is calculated using MATLAB 2016 [3,36,37] for the BCG signals. The Z-score value for each BCG signals was calculated using Equation (1) as described in [36,38,39].

(1) $$Z=\frac{Q-\mu }{\sigma }$$

In this equation, the *Q* is the value to be normalized, the mean value is represented by μ, and the standard deviation for that particular category is represented by σ.

### 3.3. Scalogram Generated Using CWT

In this study, after the BCG signals were obtained, a scalogram plot of time versus frequency represented the absolute CWT coefficients value of a signal [4,28,40]. A scalogram better identifies low frequency BCG signals or fast changing components of BCG signals when compared to a spectrogram. CWT was then used to process the 30 s BCG signals to be converted into images of RGB format. After this, we converted the RGB images into greyscale images, which were fed as input to the CNN model [41,42].

CWT is considered to be an effective analysis methodology when studying frequency information versus time. In this study, CWT was used on each of the 30 s segments, and the BCG signal was ultimately converted and represented as time frequency. As a result, the absolute values were derived for each signal segment by a previously described method [28,43,44,45]. In addition to this, scalograms were obtained by applying CWT on BCG signals using MATLAB (Wavelet toolbox). Furthermore, the Morse mother wavelet was used in CWT.

**Figure 5:**
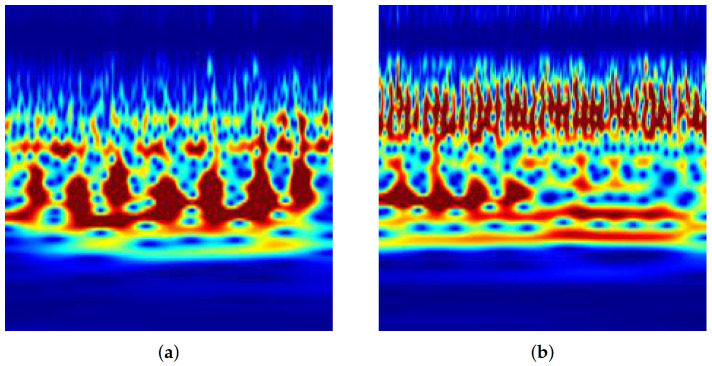
Typical scalogram signal: (**a**) HC and (**b**) HPT classes.

The obtained values were processed and adjusted to a size of 32 × 32 (the CNN pre-requisite). The CNN model was used to classify the final processed grey-scale images. Figure 5 shows the typical scalograms of the HC and HPT BCG signals.

### 3.4. Convolution Neural Network (CNN)

Three layers are generally present in a CNN model: (a) convolution layers or hidden layers, (b) pooling layers, and (c) fully connected layers. The foundation of the model is the convolutional layers [5,34,35]. Inputs are broken down with the help of modules called ‘Kernels’, each of differing data sizes, and feature maps are used for the analysis [5,34,35]. Features executed in the convolution layer paves for categorization in the other layers [34,35].

Thus, the automated extraction process is aided by the convolution and pooling layers, while the fully connected layer helps to perform the classification [34,35]. For improved diagnosis or granular data classification, the CNN model makes use of deeper layers by the kernels. While using many deep layers often leads to better learning by the machine, the complexity of computing increases with an increase in their number.

### 3.5. Proposed 2D-CNN Model

**Figure 6:**
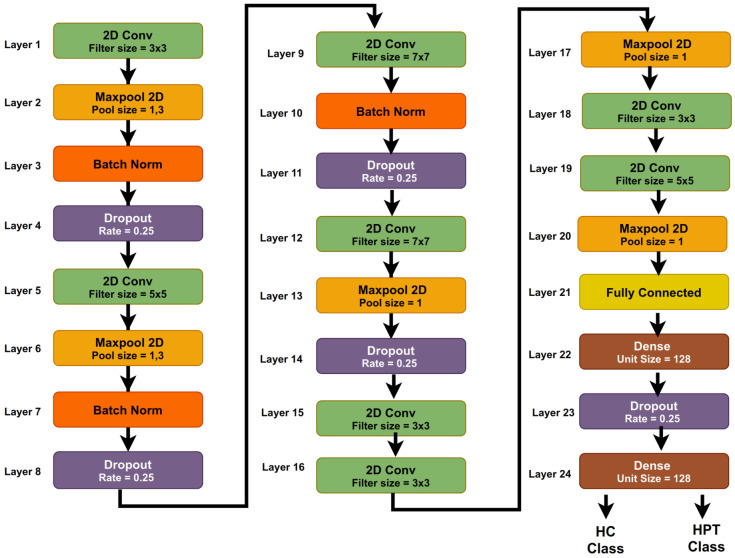
The proposed 2D CNN model.

A new 2D-CNN model was developed for the automated classification of HC and HPT classes using BCG signals. Table 2 shows the summary of the proposed 2D CNN model and the kernel sizes used in the model obtained by the trial and error method. The proposed model has 24 layers, not including the input layer. Further, the developed model includes eight 2D convolution, five 2D max-pooling, three batch normalization, five dropouts, and two dense layers. For convolution of the BCG scalogram images and max-pooling processes, the stride is set to 2 and 1, respectively. In addition to this, Figure 6 represents the proposed 2D-CNN architecture.

The input layer of the proposed model is scalograms, which are CWT of BCG signals. The 2D convolution operation is performed on the scalograms with a stride of 2 and kernel size of 3 × 3 with 64 filters. The feature maps of the input images are formed after the convolution operation. Max-pooling of size 1 with a stride of 1 is employed on these feature maps to reduce the number of neurons. In the model, batch normalization layers are employed to normalize the activation of the preceding stage in every batch. Batch normalization was added after each 2D max-pooling layer, and the kernel regularizer (L1) was used and was set to 0.001 to avoid overfitting.

Dropout layers were then added to the model to avoid additional overfitting. Dimension modifications were conducted in the flattened layer to allow the features of the preceding layer to be evaluated in dense layers. The sigmoid layer was the final layer of the proposed model and was used to perform the classification of HC and HPT classes. An Adam optimizer was used in the proposed model, with a learning rate of 0.001 and a decay rate of 0.01.

### 3.6. System Model Parameters

**Figure 7:**
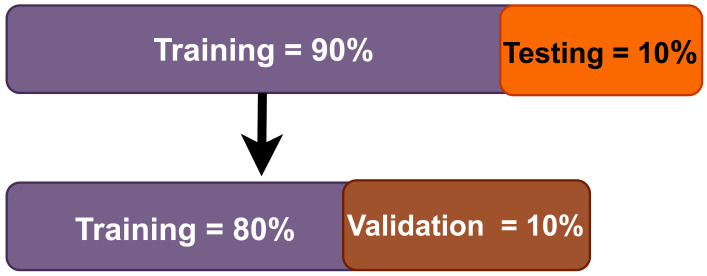
Illustration of the training, validation, and testing structures employed in this work.

Python (open source version 3.9.7) was used to implement the proposed deep-learning model. The model was built using Keras (open source version (Google) Keras 2.7.0), with Tensor-flow (open-source (created by Google) version 2.8.0) serving as the back-end. A computer with the following specifications was used to develop the model: Intel Core i7 (Intel Xenon) processor with a clock frequency of 3.5 GHz, 16 GB RAM, 4 GB NVIDIA graphics card, and a 1 TB HDD. The complete database was divided into three categories: 80%, 10%, and 10% for training, validation, and testing, respectively. Figure 7 represents the structure of the training, validation, and testing databases for the proposed model. Table 3 shows the hyper-parameters used for the proposed model.

## 4. Results

The 1D BCG signals were converted into 2D images by applying CWT on each epoch. Figure 5 shows sample scalogram images of the HC and HPT classes. These scalograms are of size 224 × 224 and are resized to 32 × 32 and then fed to the 2D-CNN model for the classification of HC and HPT classes. A total of 61,525 HPT and 71,413 HC BCG scalogram images were obtained. Hence, a total of 132,938 BCG scalogram images were used for the final analysis.

**Figure 8:**
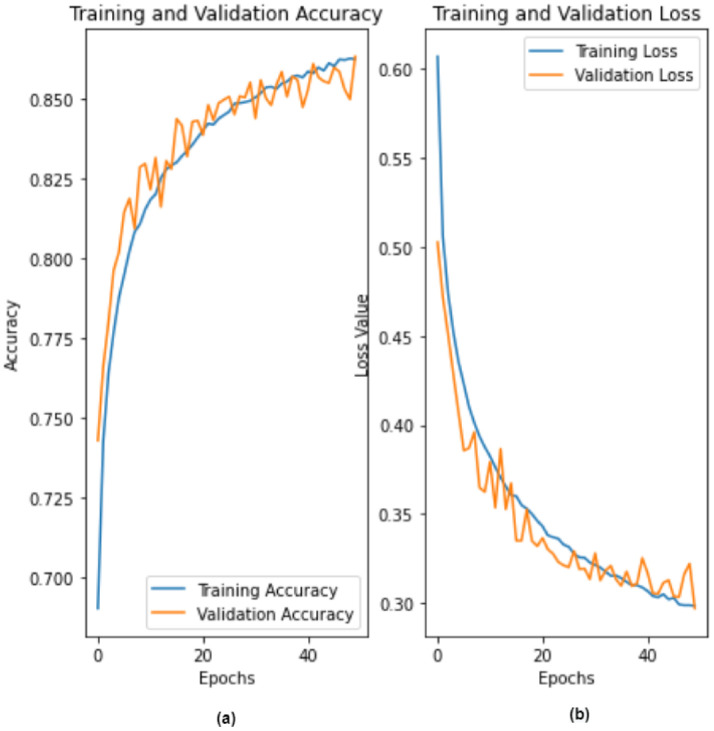
Performance graph of the proposed 2D-CNN model (**a**) accuracy (epochs vs. accuracy) and (**b**) loss (epochs vs. loss).

**Figure 9:**
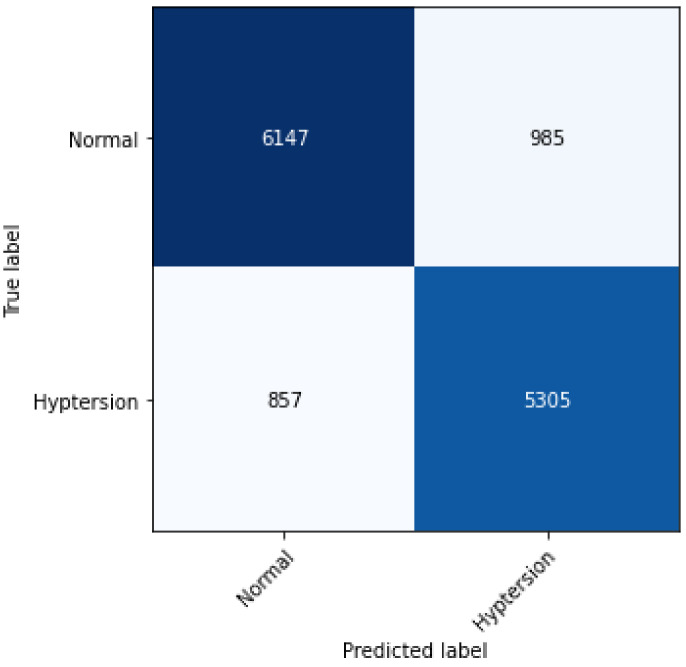
Confusion matrix obtained for the proposed model (with testing data).

**Figure 10:**
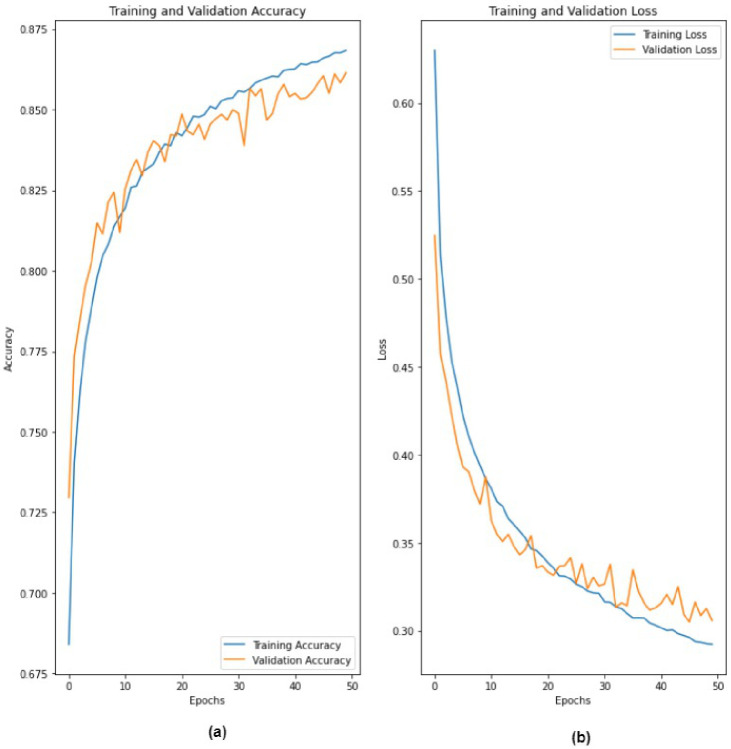
Performance graph of the proposed 2D-CNN model obtained from 10-fold cross validation methods (**a**) accuracy (epochs vs. accuracy) and (**b**) loss (epochs vs. loss).

**Figure 11:**
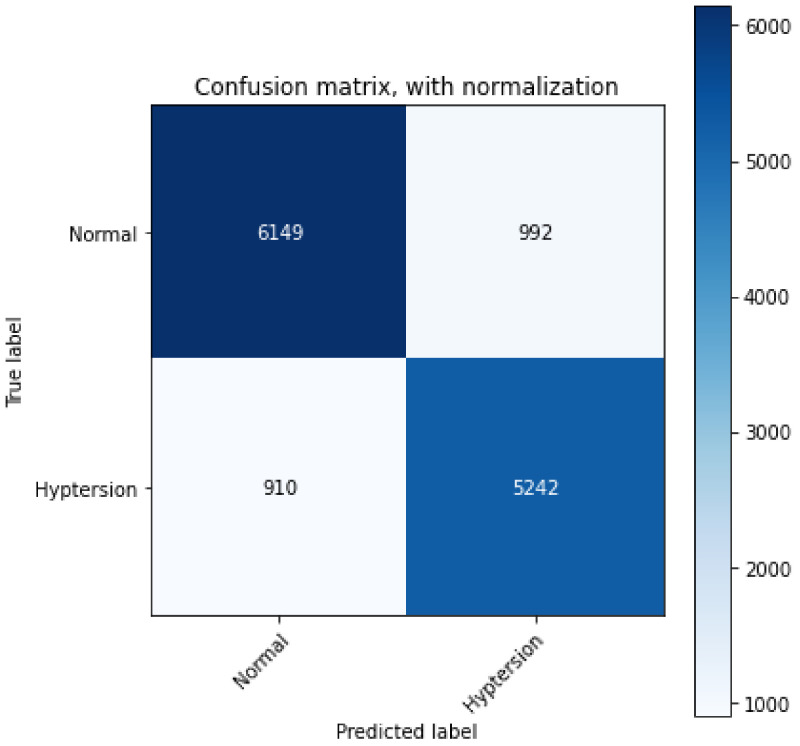
Confusion matrix obtained for the proposed model.

The proposed 2D-CNN model obtained a maximum accuracy, sensitivity, specificity, and F1-score of 86.14%, 87.6%, 84.31%, and 87%, respectively, as shown in Table 4 with a 10-fold CV method. Figure 8 depicts the proposed model’s accuracy and loss graph against 50 epochs. Figure 9 represents a confusion matrix of the testing data (with 10%). Similarly, we also validated our model with the ten-fold CV strategy. In addition to this, the results of the 10-fold methods in accuracy versus epochs, loss versus epochs, and a confusion matrix are shown in the Figure 10 and Figure 11, respectively. Furthermore, the comparison with state-of-the-art methods is shown in Table 5.

## 5. Discussion

The novelty of the present study is the use of scalogram images from BCG signals for the classification of HC and HPT classes using deep neural networks. Scalograms can help to detect the signal’s low-frequency or rapidly shifting frequency components. Further, CWT employs windows of varying widths helping to clearly discriminate low- and high-frequency information in time series [46].

However, in terms of performance, Figure 9 presents the confusion matrix of the proposed 2D-CNN model, and the diagonal values illustrate that the HC class is 86.18% correctly classified and 13.82% incorrectly classified, while the HPT class is 86% correctly classified and 14% incorrectly classified. Importantly, it is evident from Figure 9 and Figure 11 that the proposed CNN-based model does not overfit, as we employed a ten-fold cross-validation strategy to develop the model.

For the classification of BCG scalograms into HC and HPT classes, a 24-layer 2D-CNN model was proposed, and the proposed model had the best convergence and classification performance, as can be seen from the results. Deep neural networks allow for learning more complex, non-linear functions, even if they can present challenges to attain convergence. Shallow networks, on the other hand, are simpler to train, and the features collected are simplistic but may not be sufficient for accurate and robust classification [47].

Key characteristics of the CNN model include the weighted sharing of objects, thereby, reducing the training parameters. This allows for the easy implementation of complex models in neural networks and smooth training without overfitting [5]. Hence, the CNN model was the preferred model in this study.

Using the CWT filter bank and analytic Morse (3,60) wavelet with 12 voices per octave, we computed the absolute value of the wavelet coefficients for each signal segment. Table 6 shows the comparison of our results with state-of-the-art methods developed for automated HPT detection.

Using a Naive–Bayes classifier, Song et al. [25] classified BCG signals into HC and HPT classes with a 74.5% accuracy. On the other hand, Liu et al. [15] employed HRV signals to detect HPT and HC BCG signals and achieved an accuracy of 84.5% using machine-learning techniques. Unlike previous studies [15,25], the proposed method employed a deep-learning model (2D-CNN) and achieved higher performance parameters compared to previous methods.

The following are the benefits of the proposed method:

- For the classification of HPT employing BCG signals, the proposed deep-learning-based approach excelled compared to the machine-learning-based approaches employed by Liang et al. and Song et al.
- To the best of our knowledge, this is the first study to distinguish between HPT and HC subjects using direct BCG signals with a deep-learning-based model. Other previous works were developed using HRV signals.
- The proposed 2D-CNN model automatically extracted features and detected HPT accurately; hence, this is a better method than those used in [15,25].
- We segmented BCG signals into epochs of 30 s duration (rather than 1 min or longer); hence, the proposed model can be trained faster and therefore can be implemented in an integrated hardware device.
- Moreover, a CWT method was employed in the proposed method to convert BCG signals to scalograms, which contain useful information about the signal in the time–frequency domain.

From the above discussion, it is clear that the proposed method produced good results in classifying HC and HPT subjects. As a result, if BCG signals are to be processed using CNN, we suggest that they should be converted into images first by employing various transformation methods.

From the comparison Table 6, the authors soh et al. [5], Liang et al. [26,27,28], Rajput et.al. [3], and Jain et al. [1] used ECG, PPG, and HRV signals. Therefore, the limitations of ECG and PPG signals and the benefits of BCG signals are as follows. Compared to the PPG and ECG signals, the BCG can collect information about heartbeats without disturbing patients and is appropriate for long-term assessment and monitoring. Additionally, the pulse transient time of BCG signals is firmly correlated with the BP.

Therefore, the performance of the proposed work will also increase as the number of subjects increases. In addition to this, the author Song et al. [25] and Liang et al. [15] extracted HRV signals from BCG signals and obtained an accuracy of 84% and 74.5%, respectively. However, our proposed work is novel as this is the first group that employed direct BCG signals on deep-learning-based methods. The classification accuracy of 86.14% is higher than the authors [15,25].

The following are the limitations of the proposed work. The BCG signal collection using a smart mattress is one of the limitations of the current study. To collect BCG signals, the subject must be continually lying on the mattress. This method cannot investigate a subject’s physiological state while performing routine activities, and obtaining high quality signals proves a problem. To overcome this hurdle, the inclusion of other smart monitoring devices is suggested, some of which may include smart watches or smart chairs to obtain signals during daytime activities. CNN takes a long duration to train a large dataset and generally requires a GPU to accelerate the training process. As a result, this enhances the cost and complexity of the model [48].

## 6. Conclusions

HPT can be termed a chronic disease. Often, HPT leads to multi-organ ailments, along with several complications, and HPT can even lead to death. Early detection of this condition and the correct chain of treatments are the only ways to avoid fatality. A constant ambulatory blood pressure monitoring system to diagnose HPT is needed. However, it is difficult to obtain continuous readings. Hence, our present study proposed an automated diagnostic tool with a 2D-CNN model to analyze BCG signals for HPT diagnosis.

The BCG signals were converted into scalogram images using CWT and fed to the CNN model. We obtained an accuracy of 86.14% for the automated detection of HPT. Since we used a ten-fold cross-validation technique, our results are accurate and robust. In the future, we intend to validate our developed model with more data belonging to different stages (mild, moderate, and severe) of HPT from different races.

## Figures and Tables

**Table 1:** The nomenclature of BCG signal waves in terms of systolic waves (SW), diastolic waves (DW), and pre-systolic waves (PSW) [20].

Nomenclature for BCG	Description
(a) SW	The headward deflection is denoted by an H-wave. In standard recordings, the I wave is the footward deflection early in the systole cycle after the H wave. The J wave is an essential headward wave in healthy subjects; it comes late in systole and immediately follows the I wave. The K wave occurs near the end of the systole and is the footward wave that follows J.
(b) DW	The L and N waves are the two smaller headward waves that normally follow the K wave in healthy subjects; the M wave is the footward wave between them.
(c) PSW	The G wave, which comes before the H wave, is a modest footward wave.

**Table 2:** The layers employed in the proposed 2D-CNN model.

S. No.	Layer	Size of Filters	Size of Kernel	Unit Size	Parameter
1	2DConv	64	3	-	RL, STR = 2
2	Maxpool2D	-	-	1,3	STR = 1
3	Batch Norm	-	-	-	-
4	Dropout	-	-	-	RT = 0.25
5	2DConv	64	5	-	RL, STR = 2
6	Maxpool2D	-	-	1,3	STR = 1
7	Batch Norm	-	-	-	-
8	Dropout	-	-	-	RT = 0.25
9	2DConv	128	7	-	RL, STR = 2
10	Batch Norm	-	-	–	-
11	Dropout			-	RT = 0.25
12	2DConv	128	7	-	RL, STR = 2
13	Maxpool2D	-	-	1	STR = 1
14	Dropout	-	-	-	RT = 0.25
15	2DConv	256	3	-	RL, STR = 2
16	2DConv	256	3	-	RL, STR = 2
17	Maxpool2D	-	-	1	STR = 1
18	2DConv	64	3	-	RL, STR = 2
19	2DConv	64	5	-	RL, STR = 2
20	Maxpool2D	-	-	1	STR = 1
21	Flatten	-	-	-	–
22	Dense	-	-	128	RL
23	Dropout	-	-	-	RT = 0.5
24	Dense	-	-	Number of class = 2	Sigmoid

ReLu = RL; Stride = STR; and Rate = RT.

**Table 3:** Summary of the hyperparameters employed for the 2D-CNN model.

Hyperparameters	
BS	100
KP	Same
KR (L1)	0.001
OM	Adam
LRT	0.001
DR	0.01
LF	Binary cross-entropy

Batch size = BS; Kernel padding = KP; Kernel regularizer = KR; Optimizer = OM; Learning rate = LRT; Decay rate = DR; and Loss function = LF.

**Table 4:** Performance metric of the proposed method.

Measure	Highest Value
Sensitivity	0.876
Specificity	0.843
Precision	0.861
Accuracy	0.861
F1-Score	0.870

**Table 5:** Comparison with different state-of-the-art methods.

S. No	Classifier	Accuracy in %
1	SVM	84.35
2	MLP	68
3	LSTM	72
4	2D-CNN(Proposed method)	86.14

**Table 6:** Comparison of our results with existing state-of-the-art methods developed for automated HPT detection.

Authors	Classifier	Features	Signals Taken	Accuracy (in %)
Y.Song et al. [25]	Navie Bayes	Non-Linear	BCG derived HRV signal	74.5
Liu et.al. [15]	LibSVM, Decision Tree, and Naive–Bayes	Linear, Non-Linear, Time & Frequency domain	BCG derived HRV signal	84.5
Rajput et al. [3]	KNN and EBT	Sample and Wavelet entropy	ECG	99.95
Soh et al. [30]	KNN	EMD, Non-linear	ECG	97.7
Jain et al. [1]	DL, CNN	…	ECG	99.68
Liang et al. [28]	DL	…	PPG	F1-score = 92.25
Liang et al. [27]	SVM	Linear and non-linear	ECG, PPG	SEN = 94.25
Liang et al. [26]	Data mining	Time and Frequency	ECG, HRV	Acc = 84.52
Proposed method	2-D CNN (deep-learning model)	…	BCG	86.14

## Data Availability

Not applied.
